# Serum gamma-glutamyl transferase, a marker of alcohol intake, is associated with telomere length and cardiometabolic risk in young adulthood

**DOI:** 10.1038/s41598-021-91987-6

**Published:** 2021-06-11

**Authors:** Esmée M. Bijnens, Catherine Derom, Evert Thiery, Dries S. Martens, Ruth J. F. Loos, Steven Weyers, Tim S. Nawrot

**Affiliations:** 1 Centre for Environmental Sciences, Hasselt University, Agoralaan Building D, 3590 Diepenbeek, Belgium; 2 Department of Human Structure and Repair, Ghent University Hospital, Corneel Heymanslaan 10, 9000 Ghent, Belgium; 3 Centre of Human Genetics, University Hospitals Leuven, Herestraat 49, 3000 Leuven, Belgium; 4 Department of Neurology, Ghent University Hospital, Corneel Heymanslaan 10, 9000 Ghent, Belgium; 5 The Genetics of Obesity and Related Metabolic Traits Program, The Charles Bronfman Institute for Personalized Medicine, The Mindich Child Health and Development Institute, The Icahn School of Medicine at Mount Sinai, 1468 Madison Ave, New York, USA; 6 Department of Public Health, Leuven University (KU Leuven), Kapucijnenvoer 35, 3000 Leuven, Belgium

**Keywords:** Molecular biology, Biomarkers, Risk factors

## Abstract

Studies based on self-reported alcohol consumption and telomere length show inconsistent results. Therefore, we studied the association between gamma-glutamyl transferase (GGT), a widely used biomarker of alcohol intake, and telomere length. The possible health relevance in young adulthood was explored by investigating cardiometabolic risk factors. Mixed modelling was performed to examine GGT and alcohol consumption in association with telomere length in buccal cells of 211 adults between 18 and 30 years old of the East Flanders Prospective Twin Survey. In addition, we investigated the association between GGT and cardiometabolic risk factors; waist circumference, systolic blood pressure, fasting glucose, HDL cholesterol, and triglycerides. Although we did not observe an association between self-reported alcohol consumption and telomere length, our results show that a doubling in serum GGT is associated with 7.80% (95% CI − 13.9 to − 1.2%; p = 0.02) shorter buccal telomeres, independently from sex, chronological age, educational level, zygosity and chorionicity, waist-to-hip ratio and smoking. The association between GGT was significant for all five cardiometabolic risk factors, while adjusting for age. We show that GGT, a widely used biomarker of alcohol consumption, is associated with telomere length and with risk factors of cardiometabolic syndrome, despite the young age of this study population.

## Introduction

Telomeres are the ends of the chromosomes and consist of TTAGGG tandem DNA-sequence repeats. They form a nucleoprotein complex and protect the chromosomes from degradation, which would eventually lead to loss of genetic information^1^. Telomeres shorten due DNA replication and are suggested to be particularly sensitive to oxidative stress^2^, as this can cause cleavage specifically at the polyguanosine sequence in the telomeric region^3^. GGG‐specific DNA damage may play an important role in increasing the rate of telomere shortening, which might impact aging and chronic diseases^4^. Telomere length is associated with several life-style factors in adults, including smoking^5,6^, stress^7,8^, diet^9^, education^10^, obesity^11^, and air pollution^12,13^. Some of these factors, such as maternal pre-pregnancy BMI and prenatal air pollution exposure, even explain the variance of telomere length at birth^14–16^. Telomere length is proposed as a biomarker of ageing and implicated in the pathology of several age-related chronic diseases, such as cardiovascular disease, osteoporosis, Alzheimer’s disease, cancer, and mortality^17,18^.

Epidemiologic studies investigating the association between alcohol consumption and telomere length show inconsistent results. Although some studies found no association^19–23^, a few studies^24,25^ suggest that heavy drinking is associated with shorter telomeres. A limitation of these studies is that alcohol consumption is based on self-reported intake and respondents may overestimate or underestimate the amount of alcohol consumed. Therefore, it is interesting to explore the association between markers of alcohol intake and telomere length. Gamma-glutamyl transferase (GGT) is a widely used biomarker of alcohol intake^26^. GGT maintains adequate levels of intracellular glutathione, a major antioxidant, to protect cells against oxidants which are produced during normal alcohol metabolism^26^. The primary role of cellular GGT is to degrade extracellular glutathione at the cell membrane into amino acids which can be used for the synthesis of intracellular glutathione^26^. Hence, elevated serum GGT might be an marker for oxidative stress^27^.

Emerging evidence demonstrates that telomere biology is linked to numerous age-related diseases^17,18^. However, further research is needed to investigate the causal involvement of telomere biology and to expose the mechanisms contributing to the development of these diseases. As telomere length, GGT is also strongly associated with risk of cardiovascular disease^28^, metabolic syndrome^29,30^, and mortality^31,32^. Although the association between GGT and telomere length is barely studied^33,34^. Advanced understanding of their interrelation already in young adulthood may be relevant to provide new insights on the underlying pathways of disease development. We hypothesize that elevated serum GGT would identify individuals with shorter telomere length. Furthermore, to explore in young adults the possible health implications of alcohol consumption and oxidative stress, indicated by high serum GGT concentrations, we investigate the association between GGT and cardiometabolic risk factors and the role of biological ageing.

## Results

### Characteristics of the study population

Our study population comprises 211 individuals; 69.2% (n = 146) of the participants included both twins from each twin pair, whereas the remaining 30.8% (n = 65) only had one participating twin from each twin pair (Table 1). Our sample comprised of 104 (49.3%) men and 107 (50.7%) women with a mean (SD) age of 22.8 (3.2) years. Our analysis included 68 (32.2%) dizygotic twins, 69 (32.7%) monozygotic-dichorionic twins and 74 (35.1%) monozygotic-monochorionic twins. The average number of reported alcohol consumptions per week (SD) was 5.4 units (7.9) and the median (IQR) serum GGT concentration was 16 U/L (12–20) with a minimum of 7 U/L and a maximum of 110 U/L. The mean T/S (SD) was 1.04 (0.28) in adult buccal cells. We observed a significant intra-pair correlation in serum GGT between twin 1 and twin 2 (r = 0.68, p < 0.001, n = 73). The correlation was significantly stronger (p = 0.01) in monozygotic twins (r = 0.85, p < 0.001, n = 47) compared with dizygotic twins (r = 0.49, p = 0.01, n = 26), suggesting a role for genetic influences (heritability of 72%). The correlation in relative adult telomere length in buccal cells was similar (p = 0.99) in monozygotic and dizygotic twins (Supplement Fig. 1).

**Table 1:** Study population characteristics.

Characteristic	Missing	Value
**Adulthood**		**n = 211**
Age, years		22.8 ± 3.2
Birth year		1976 ± 3.2
Sex		
Male		104 (49.3)
Female		107 (50.7)
Body mass index, kg/m^2^		21.5 ± 2.7
Waist-to-hip ratio		0.78 ± 0.07
Smokers, n		67 (31.8)
Alcohol consumptions per week, units		5.4 ± 7.9
Medication use (possibly associated with GGT) 14 days prior, n		12 (5.7)
Gamma-glutamyl transferase, U/L; median (IQR)		16 (12–20)
Telomere length		
Birth	72	1.30 ± 0.61
Young adulthood		1.04 ± 0.28
Education twin		
Low		67 (31.7)
High		144 (68.3)
Zygosity-chorionicity		
Dizygotic–dichorionic		68 (32.2)
Monozygotic–dichorionic		69 (32.7)
Monozygotic–monochorionic		74 (35.1)
Complete-pair in final study		
One twin		65 (30.8)
Both twins		146 (69.2)
**Maternal**		**n = 138**
Socioeconomic status: maternal education	8	
Low		53 (40.8)
Middle		30 (23.1)
High		47 (36.1)

Data presented are means ± standard deviation or number (percentage).

### Gamma-glutamyl transferase and telomere length

**Figure 1:**
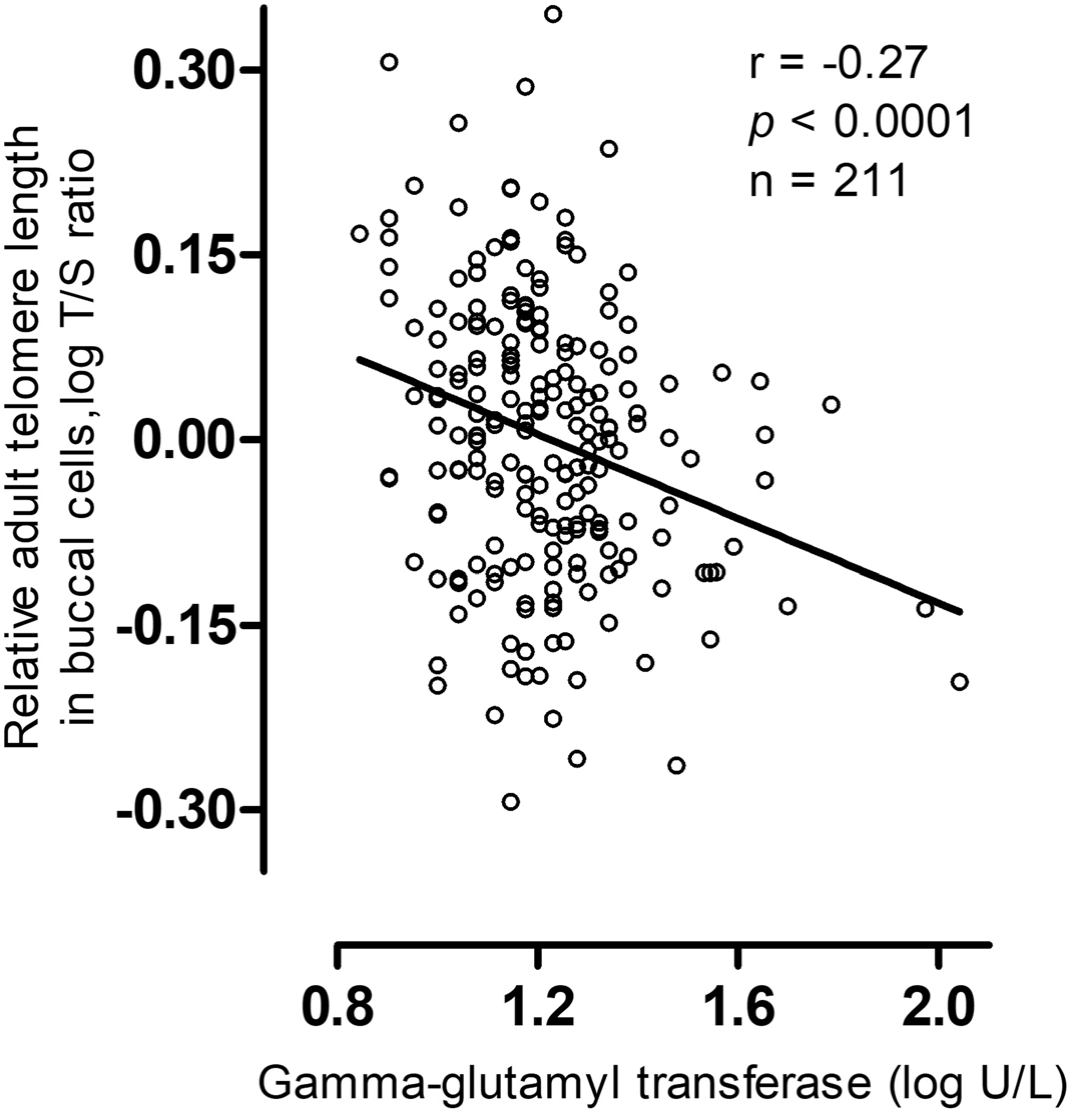
Gamma-glutamyl transferase in association with relative adult telomere length in buccal cells. The figure was plotted using GraphPad Prism version 5.00 (https://www.graphpad.com/).

**Figure 2:**
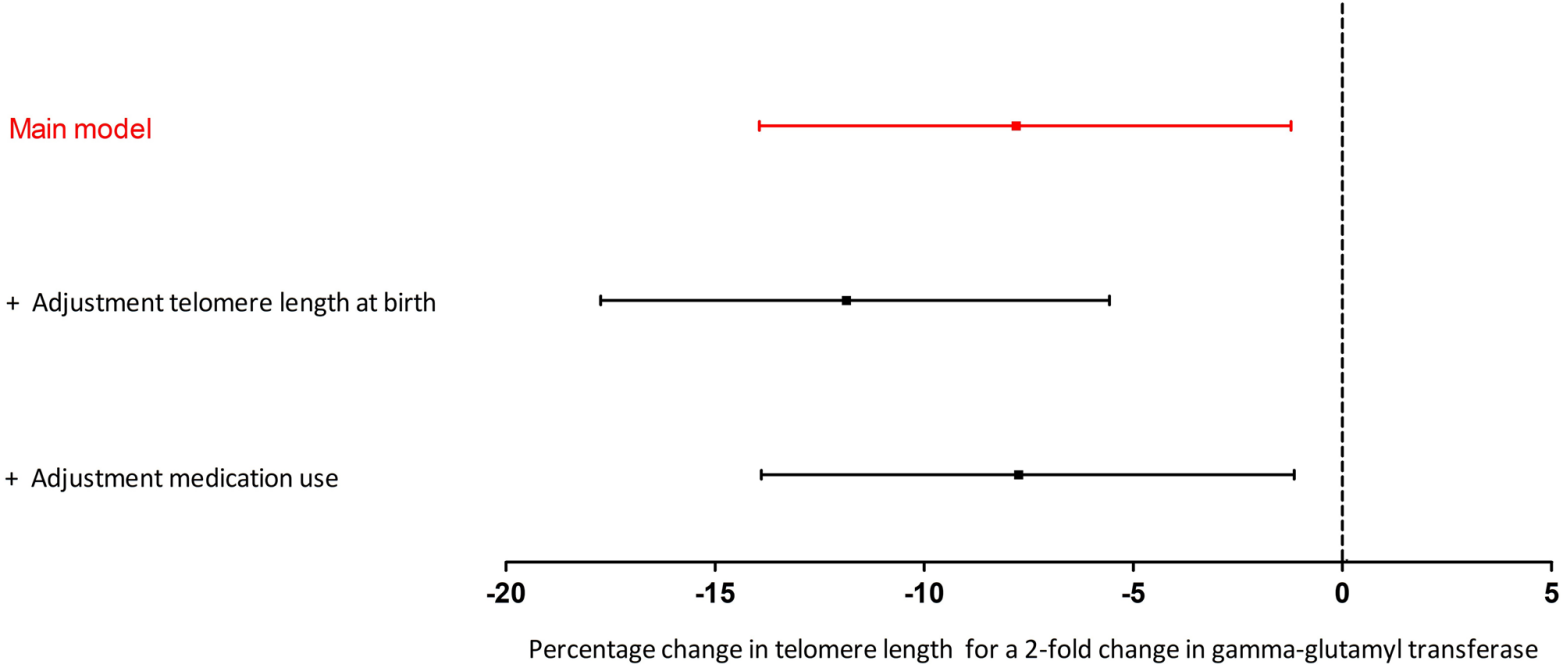
Gamma-glutamyl transferase in association with relative adult telomere length in buccal cells. The main model is adjusted for sex, age (linear and quadratic), zygosity and chorionicity, education twin, maternal education, waist-to-hip ratio, and smoking (n = 200). The second model is additionally adjusted for telomere length at birth (n = 137). The third model is additionally adjusted for medication use, possibly associated with gamma-glutamyl transferase, during the last 14 days prior to the examination (n = 200). The figure was plotted using GraphPad Prism version 5.00 (https://www.graphpad.com/).

Serum GGT correlated inversely with telomere length in young adulthood (r = − 0.27, p < 0.0001) as shown in Fig. 1. A doubling in GGT is associated with 7.80% shorter telomeres (95% CI − 13.9 to − 1.2; p = 0.02) when adjusting for sex, age, zygosity and chorionicity, education twin, maternal education, waist-to-hip ratio and smoking (Fig. 2). As telomere length is highly variable at birth we additionally adjusted for telomere length at birth. In this analysis accounting for telomere length at birth, we show that a doubling in serum GGT was associated 11.86% shorter telomeres (95% CI − 17.7 to − 5.6; p = 0.001). The association between GGT and telomere length remained significant after adjusting for cardiometabolic risk factors. Finally, after additional adjustment for medication use possibly associated with GGT 14 days prior to examination, the association between GGT and telomere length remained significant (Fig. 2).

The number of alcohol consumptions per week (in units) was positively correlated with GGT (r = 0.29, p < 0.0001) (Supplement Fig. 2). However, we observed no correlation (r = − 0.03, p = 0.70) between the number of units alcohol consumptions per week and telomere length. Although we noted a negative correlation (r = − 0.15, p = 0.03) between the number of years of drinking and telomere length in young adulthood, this did not remain significant after adjusting for age.

### Gamma-glutamyl transferase and telomere length in association cardiometabolic risk

We observed a significant association between GGT and cardiometabolic risk factors. After adjusting for age, a doubling in GGT is associated with a 0.10 mmol/l increase in fasting glucose (95% CI 0.01 to 0.18; p = 0.03), a 5.52 cm increase in waist circumference (95% CI 4.0 to 7.1; p ≤ 0.0001), a 11.11 mmol/l increase in triglycerides (95% CI 2.6 to 19.6; p = 0.01), a 4.91 mmol/l decrease in HDL cholesterol (95% CI − 8.7 to − 1.1; p = 0.01), a 5.03 mmHg increase systolic (95% CI 2.2 to 7.8; p = 0.0008) and a 2.37 increase diastolic blood pressure (95% CI 0.25 to 4.5; p = 0.03). We did not observe a significant association between telomere length and cardiometabolic risk factors after adjusting for age. Only a doubling in telomere length was significantly associated with a − 0.13 mmol/l decrease in glucose (95% CI − 0.25 to − 0.003; p = 0.05). These results are shown in Table 2.

**Table 2:** Gamma-glutamyl transferase and telomere length in association with risk markers of cardiometabolic syndrome.

	Change	95% CI	P-value
**Gamma-glutamyl transferase log (twofold change)**			
Glucose, mmol/l	0.10	0.01 to 0.18	0.03
Waist circumference, cm	5.52	4.0 to 7.1	< 0.0001
Triglycerides, mmol/l	11.11	2.6 to 19.6	0.01
HDL cholesterol, mmol/l	− 4.91	− 8.7 to − 1.1	0.01
Systolic blood pressure, mmHg	5.03	2.2 to 7.8	0.0008
Diastolic blood pressure, mmHg	2.37	0.25 to 4.5	0.03
**Telomere length (twofold change)**			
Glucose, mmol/l	− 0.13	− 0.25 to − 0.003	0.05
Waist circumference, cm	− 0.76	− 3.2 to 1.7	0.54
Triglycerides, mmol/l	3.47	− 9.7 to 16.6	0.61
HDL cholesterol, mmol/l	1.88	− 3.5to 7.3	0.49
Systolic blood pressure, mmHg	− 3.49	− 7.7 to 0.7	0.11
Diastolic blood pressure, mmHg	− 2.08	− 5.2 to 1.0	0.19

Adjusted for age.

## Discussion

We investigated the association between serum gamma-glutamyl transferase, a biomarker of alcohol intake and cardiometabolic risk factors, and telomere length in 211 young adults. Although we did not observe an association between self-reported alcohol consumption and telomere length, we did observe that serum GGT has a negative association with telomere length in buccal cells. A doubling in serum GGT is associated with 7.80% shorter telomeres. This association remains significant after additional adjustment for telomere length at birth. Furthermore, adjusting for the use of specific medication^35^, which could potentially elevate GGT levels, did not influence the results.

Compared with our results, a recent study in 205 individuals in South Africa noted a significant negative correlation between leukocyte telomere length and GGT (r = − 0.16, p = 0.03)^34^. In contrast to our findings, no associations between telomere length and indicators of oxidative stress including GGT were observed in a study in 143 elderly Dutch men and 109 Greek elderly men^33^. Even though studies on GGT and telomere length are limited, a previous study in 372 persons already observed an association between the glutathione cycle and leukocyte telomere length^36^. They found a negative correlation between blood gamma-glutamyl amino acids and telomere length. These metabolites indicate increased oxidative stress due to alterations in the glutathione metabolism.

Therefore, oxidative stress may be a possible underlying mechanism between GGT and telomere length. GGT maintains the cytoplasmic homeostasis of glutathione, an important antioxidant which is crucial for detoxification of reactive oxygen species (ROS) as well as other toxic compounds^27^. Telomeres are especially sensitive to oxidative stress due to their triple-G containing structure and also have a low efficiency of DNA damage repair. As a result, telomeres containing oxidative damage will become successively shorter in each round of replication and the sequence beyond the damage will be lost^2,37^.

We show a positive correlation between GGT and the number of alcohol consumption per week. Our findings support GGT as a biomarker of alcohol use^26^. The well-known elevation of serum GGT after alcohol consumption could be a consequence of the increases the production of ROS after chronic and acute alcohol consumption^27^. Previous studies investigating the association between alcohol consumption, based on self-reported intake, and telomere length show inconsistent results. Although some studies found no association^19–23^, various other studies^24,25^ suggest that heavy drinking is associated with shorter telomeres. We did not observe an association between reported alcohol consumption and telomere length in our study population. This may be due to underreporting and biases regarding self-reported alcohol use, attenuating the true magnitude of the association.

Our findings have the potential to be relevant for public health since GGT is more than an indicator of alcohol consumption, GGT is strongly associated with risk of cardiovascular disease^28^ and metabolic syndrome^29,30^. A 2014 meta-analysis by Kunutsor et al. of 29 cohort studies showed an association between GGT and cardiovascular disease with an adjusted relative risk of 1.23 (1.16–1.29) per 1 standard deviation change in log GGT^28^, whereas a meta-analysis of 10 prospective cohort studies comprising 6595 incident metabolic syndrome cases, observed an association between GGT and metabolic syndrome with a relative risk of 1.88 (1.49–2.38) in the top versus the bottom thirds of baseline GGT^29^.

Although the number of metabolic syndrome cases is very low (n = 6) in our young study population (age between 18 and 30 years), we already observe that GGT is significantly associated with cardiometabolic risk factors defined by the National Cholesterol Education Program (NCEP) Adult Treatment Panel III (ATP III) criteria for metabolic syndrome^38^. These include waist circumference, systolic blood pressure, fasting glucose, HDL cholesterol, and triglycerides^38^. Possible underlying processes of metabolic syndromes that have been postulated to be related to GGT are inflammation, oxidative stress, and insulin resistance^30^. Contrary to GGT, telomere length was not associated with cardiometabolic risk factors in this study population. A previous cross-sectional study including older participants, aged 18–65 years, showed that shorter telomeres are significantly associated with the presence of metabolic syndrome and all metabolic risk factors, except blood pressure^39^. Elucidating the underlying mechanism of both telomere length and GGT already in young adulthood will contribute to the understanding of the development of disease later in life. However, further research with a longitudinal study design, is needed to investigate whether GGT in young adulthood is a predictor of disease development later in life and to explore if GGT is directly associated with cardiometabolic risk or indirectly via telomere length. The answer to this question has the potential to provide opportunities in prevention of cardiovascular disease and metabolic syndrome.

It is an advantage that the results were obtained using mixed models since this method prevents false positive associations due relatedness structure between twin members and increases in power obtained through the application of a correction that is specific to this structure^40^. However, there are limitations to this study. First, DNA was collected via non-invasive buccal swabs. Although the absolute length of the telomeres might differ between tissues, within an individual telomere length is highly correlated between buccal cells and blood^41,42^. However, disadvantages include the lower quality of the DNA isolated from buccal swabs^43^ and the risk that poor oral hygiene or infection during collection might alter the oral cell composition due to infiltration of immune cells with a different telomere profile than buccal cells^44^. Compared to leukocyte telomeres which might be affected by a more diverse cell population in blood, buccal cell telomeres are less influenced by different types of cells^44^. A second limitation of our study is that no data is available on other markers of alcohol consumption, such as aspartate aminotransferase (AST) and alanine aminotransferase (ALT). This would be an enhancement because GGT levels likely vary due to medications use, liver disease, and other etiological changes. Third*,* it is a limitation that our observations are made in a small study population as this may lead to a higher variability. Lastly, although we adjusted for potential major confounders that may affect both GGT and telomere length, we acknowledge that the main results cannot ascertain causality. Therefore, although we have hypothesised that variation in GGT influences telomere length, and undertaken the analyses accordingly, we cannot exclude the possibility that the association is confounded by factors, including genetic variants, that independently influence both telomere length and GGT, nor can we exclude reverse causation occurring when people with short telomeres are more prone to consuming alcohol and have higher GGT levels than people with longer telomeres.

## Conclusions

We observed a strongly negative association between serum GGT and telomere length in buccal cells. Our results suggest that GGT, a biomarker of alcohol consumption, is a better predictor of telomere length than self-reported alcohol consumption. We suggest that the underlying mechanism may be oxidative stress. Despite the young age of our study population, we observed an association between GGT and indicators of cardiometabolic disease.

## Materials and methods

### Subjects

The twins in our study participated in a prenatal programming study^45^ comprising 424 twin pairs (804 individuals), part of The East Flanders Prospective Twin Study (EFPTS)^46^. The EFPTS, a population based register of multiple births in the province of East Flanders (Belgium), collects perinatal data and examines the placenta at birth since 1964. Telomere length in buccal cells was available for 214 individuals as described previously^47^. We excluded 3 participants because missing data on GGT (2) and on alcohol consumption (1). The present study sample consist of 211 young adults, born between 1969 and 1981. Written informed consent was obtained from all participants, and ethical approval was given by the Ethics Committee of the Faculty of Medicine of the Katholieke Universiteit Leuven. This study has been carried out according to the Helsinki declaration.

### Data collection

Biometric and laboratory measurements of adult twins were obtained at the research centre during a 2-h morning session between February 1997 and April 2000^45^. All methods were performed in accordance with relevant regulations and all measurements were carried out following standardized guidelines. In fasting blood samples, gamma-glutamyl transferase was measured using an Olympus AU600 Auto-Analyzer (Kyoto, Japan) and plasma glucose, HDL cholesterol, and triglycerides were measured using an auto‐analyzer (AU600; Olympus). Anthropometric measurements were performed by two trained researchers according to standardized procedures. Body mass index was calculated as body mass (in kg) divided by squared height (in m). Standing height was measured with a Harpenden fixed stadiometer (Holtain Ltd, United Kingdom) to the nearest 0.1 cm and body mass on a balance scale (SECA, Germany) to the nearest 0.1 kg. Waist and hip circumference were measured with a flexible steel tape to the nearest 0.1 cm. Waist circumference was taken between the costal margin and the iliac crest, and hip circumference at the widest part of the hips, generally at the level of the greater trochanters. The waist-to-hip ratio was calculated as the ratio of the circumference of the waist to that of the hips. Blood pressure was measured on the right arm in triplicate by sphygmomanometry and auscultation (Korotkoff phases I and V) in sitting position. The reported blood pressure is the average of the 3 measurements. Cardiometabolic risk factors were based on the National Cholesterol Education Program (NCEP) Adult Treatment Panel III (ATP III) criteria for metabolic syndrome and include waist circumference, systolic blood pressure, fasting glucose, HDL cholesterol, and triglycerides^38^.

The twins completed questionnaires to obtain information on smoking status, alcohol use, disease and medication use. Current smoking was expressed as yes/no and current alcohol use was analyzed as units per week. Each unit was equivalent to approximately 10–12 g alcohol intake. We obtained the international classification of disease, 10th revision (ICD10) codes for the disease status of the participants but no one was diagnosed with liver disease. Medication use possibly associated with increased GGT (such as anticonvulsants, antacids, retinoids, and antidepressants) during the last 14 days prior to the examination was expressed as yes/no. Zygosity was determined at birth by sequential analysis based on sex, choriontype, blood group determined on umbilical cord blood, placental alkaline phosphatase and, since 1982, DNA fingerprints^48^. After DNA-fingerprinting, a zygosity probability of 99.9% was reached.

Parental educational level as a proxy of socioeconomic status was categorized into three groups according to the Belgian education system, namely as no education or primary school, lower secondary education, and higher secondary education and tertiary education. Education level of the twin was categorized into two groups, higher secondary education or lower and tertiary education.

### Telomere length measurements

Mouth swabs were taken and DNA was extracted with the QIAamp DNA micro kit (Qiagen, Venlo, The Netherlands). DNA purity and concentration were assessed using the Nanodrop 1000 spectrophotometer (Isogen Life Science, Belgium). The methods for telomere length measurement in buccal cells have been previously described^47^. In brief, using an adapted quantitative real-time PCR method, relative telomere length was determined as the ratio of telomere sequence repeats to a single copy nuclear control gene, 36B4 (acidic ribosomal phosphoprotein P0). Each PCR reaction was performed in triplicate and three non-template controls as well as six inter-run calibrators were included on each 384-well plate. All samples were analyzed with the 7900HT Fast Real-Time PCR system (Life Technologies). After thermal cycling, raw data was collected and processed. The relative average telomere length was calculated as the ratio of the cycle threshold value of telomere sequence repeat to single copy gene (T/S) in the study participants compared with that of the averaged T/S value for the study population using the qBase software (Biogazelle, Zwijnaarde, Belgium). The program uses modified software based on the classic comparative CT method and takes the reference gene into account and uses inter-run calibration algorithms to correct for run-to-run differences^49^. All samples were analyzed in triplicate and included in the study when the difference in quantification cycle (Cq) value was less than 0.50. The coefficients of variation within triplicates of the telomere and single copy gene assay were 0.48% and 0.31%, respectively. The inter-assay coefficient of variation was 7.38%. We observed no correlation between the coefficient of variation with the mean buccal telomere length of the triplicates (r = 0.10; P = 0.17). The methods for telomere length measurement in placental tissue is similar to the method described above. Except HBG (human beta-globin) is used as single copy control gene instead of 36B4 and triplicate values were only included when the difference in Cq was less than 0.30.

### Statistical analyses

We used SAS software, version 9.4 (SAS Institute, Cary, NC), for data management and statistical analyses. All reported P values are two-sided and were considered statistically significant when P < 0.05. After inspection of the distribution of all variables, measures of GGT and telomere length were log_10_-transformed. To assess the unadjusted relations between GGT and telomere length and alcohol consumption, the Pearson correlation coefficients were determined. Mixed modelling was performed to investigate buccal telomere length in young adulthood in association with GGT. The twins were analyzed as individuals using a multilevel regression, which accounts for relatedness between members of a twin pair by adding a random intercept to the model. The variance–covariance structure was allowed to differ between the three zygosity and chorionicity groups, including dizygotic dichorionic, monozygotic dichorionic and monozygotic monochorionic. Mixed modelling was performed adjusted for covariates selected a priori, namely sex, age (linear and quadratic), zygosity and chorionicity, education twin, maternal education, waist-to-hip ratio and smoking. To capture potential non-linear effects of year of age, the quadratic terms of this variable was added. In addition, we performed sensitivity analyses with additional adjustment for telomere length at birth and the use of medication possibly associated with elevated GGT. In addition, we also investigated the association between GGT and risk factors of cardiometabolic syndrome, while adjusting for age.

Finally, twins were studied as pairs, we analysed the correlation between GGT in twin 1 and twin 2 for monozygotic and dizygotic twins separately. This was also repeated for telomere length. Since monozygotic twins are genetically identical, whereas DZ twins share half of their genes, a greater within-pair similarity in MZ twins than in DZ twins reflects genetic influences. If this is the case, the heritability (h) of the GGT and telomere length can be estimated form twice the difference between MZ and DZ correlations. Using the twin design, the relative influence of genetic and environmental factors on birth can be estimated. The significance level for the difference in intra-pair correlation coefficient between groups is tested with a Fisher Z transformation.

## Supplementary Information

Supplementary Figures.
