# Supplementary material for: Serum gamma-glutamyl transferase, a marker of alcohol intake, is associated with telomere length and cardiometabolic risk in young adulthood
Source: Sci Rep. 2021 Jun 11;11:12407. doi: 10.1038/s41598-021-91987-6 (PMC8196210; doi:10.1038/s41598-021-91987-6)
Supplement: Supplementary file 1 — Supplementary Figures. [file 41598_2021_91987_MOESM1_ESM.docx]

**Serum gamma-glutamyl transferase, a marker of alcohol intake, is associated with telomere length and cardiometabolic risk in young adulthood**

**Esmée M Bijnens^1,2 *^, PhD; Catherine Derom^2,3^, PhD; Evert Thiery^4^, MD PhD; Dries S Martens^1^, PhD; Ruth JF Loos^5^, MD PhD; Steven Weyers^2^, MD PhD; Tim S Nawrot^1,6^, PhD**

1. Centre for Environmental Sciences, Hasselt University, Agoralaan building D, 3590 Diepenbeek, Belgium
2. Department of Human Structure and Repair, Ghent University Hospital, Corneel Heymanslaan 10, 9000 Ghent, Belgium
3. Centre of Human Genetics, University Hospitals Leuven, Herestraat 49, 3000 Leuven, Belgium
4. Department of Neurology, Ghent University Hospital, Corneel Heymanslaan 10, 9000 Ghent, Belgium
5. The Genetics of Obesity and Related Metabolic Traits Program, The Charles Bronfman Institute for Personalized Medicine, The Mindich Child Health and Development Institute, The Icahn School of Medicine at Mount Sinai, 1468 Madison Ave, New York, United States
6. Department of Public Health, Leuven University (KU Leuven), Kapucijnenvoer 35, 3000 Leuven, Belgium

*** Corresponding author:** dr. Esmée Bijnens, Agoralaan Building D, 3590 Diepenbeek, Belgium.

Email: esmee.bijnens@uhasselt.be. Tel: +3211268365.

| **All (n=73 pairs)** | **Dizygotic twins (n=26 pairs)** | **Monozygotic twins (n=47 pairs)** |  |  |
| --- | --- | --- | --- | --- |
| 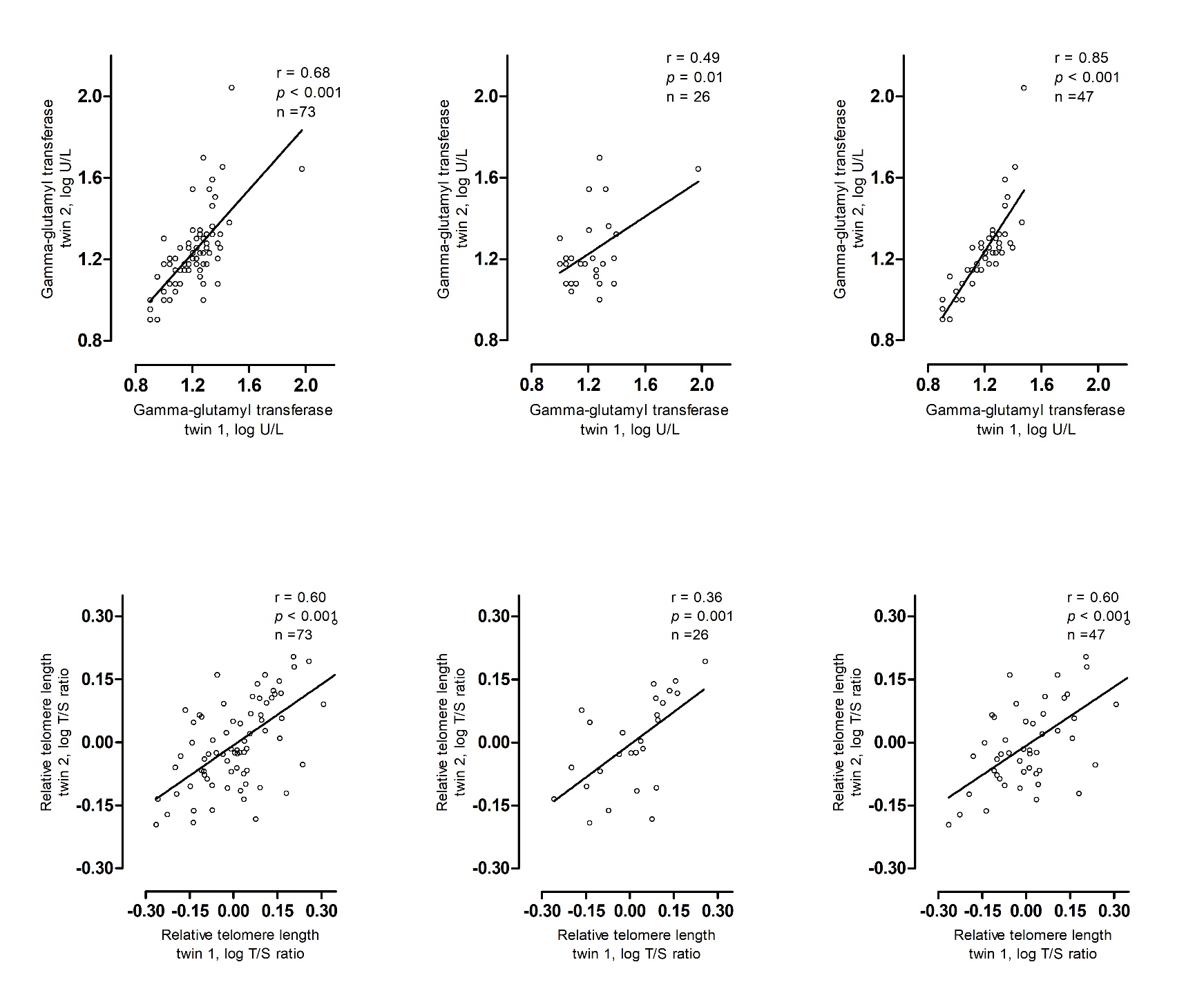 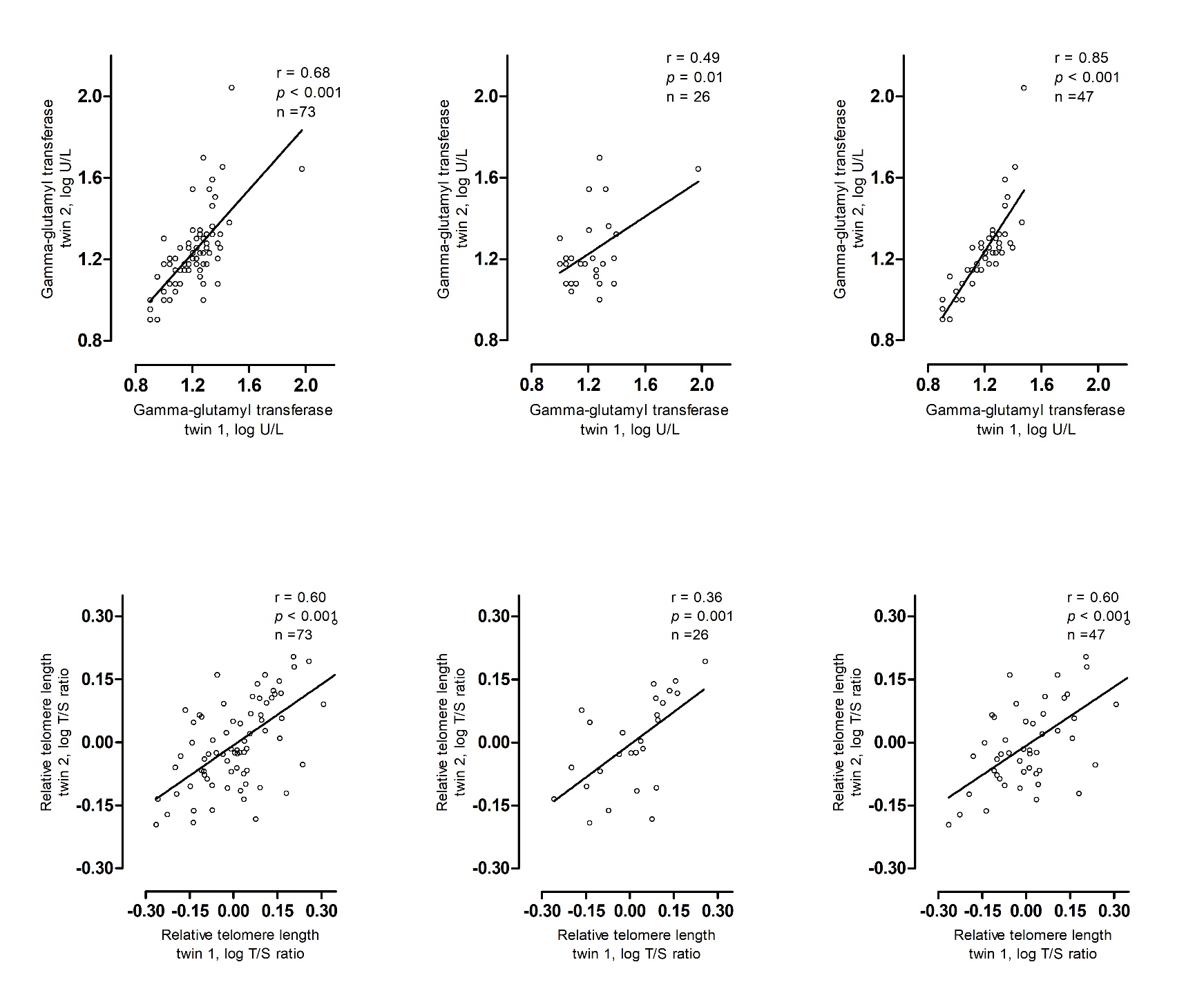 | | | | |
| **Supplement figure 1** Intra-pair correlation between twin 1 and twin 2 in log gamma-glutamyl transferase and relative adult telomere length in buccal cells in all twins, dizygotic twins and monozygotic twins. The intra-pair correlation in log gamma-glutamyl transferase is significantly (p-value=0.01, Fisher Z transformation) different between monozygotic and dizygotic twins. The intra-pair correlation coefficients for telomere length do not differ (p-value = 0.99). | | | | |


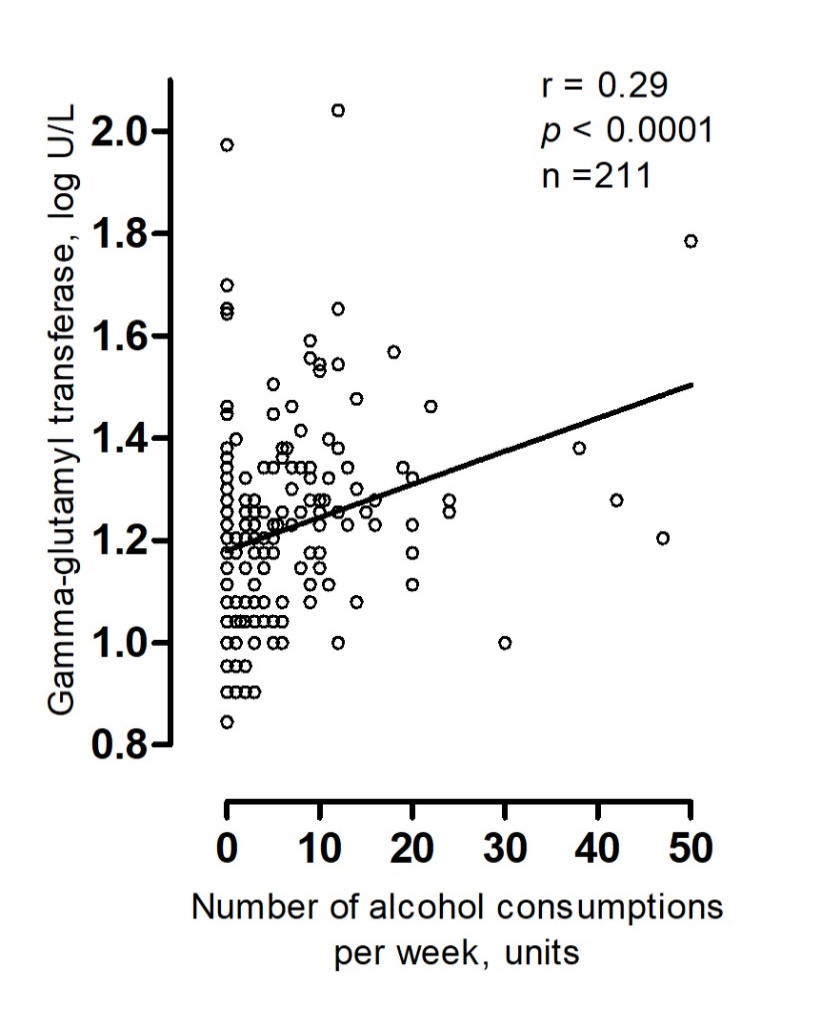


**Supplement figure 2** Number of alcohol consumptions per
week in association with gamma-glutamyl transferase (log).
